# Intraepithelial γδ T Cells Remain Increased in the Duodenum of AIDS Patients Despite Antiretroviral Treatment

**DOI:** 10.1371/journal.pone.0029066

**Published:** 2012-01-04

**Authors:** Dag E. Nilssen, Per Brandtzaeg

**Affiliations:** 1 Laboratory for Immunohistochemistry and Immunopathology (LIIPAT), Centre for Immune Regulation (CIR), University of Oslo, and Department of Pathology, Oslo University Hospital, Rikshospitalet, Oslo, Norway; 2 Department of Medicine, Akershus University Hospital, Lørenskog, Norway; University of California San Francisco, United States of America

## Abstract

Intraepithelial lymphocytes (IELs) bearing the γδ T-cell receptor are a unique intestinal subset whose function remains elusive. Here, we examine how they behave in AIDS and during various regimens of antiretroviral treatment in order to obtain mechanistic insight into their adaptive or innate functional *in vivo* properties. IELs were studied by multimarker two-colour immunofluorescence *in situ* staining. Consecutive duodenal biopsies were obtained from advanced infection-prone HIV^+^ patients (*n* = 30). The systemic adaptive immune status was monitored by determining T-cell subsets and immunoglobulins in peripheral blood. The γδ IEL ratio (median 14.5%, range 1.5–56.3%) was significantly increased (p<0.02) compared with that in clinically healthy HIV^−^ control subjects (*n* = 11, median 2.8%; range 0.3–38%), although the number of γδ IELs per mucosal length unit (U) only tended to be increased (4.0/U in HIV^+^ *versus* 3.2/U in HIV^−^subjects). Notably, the total number of CD3^+^ IELs was significantly reduced in AIDS (p<0.0001, 39.6/U in HIV^+^ *versus* 86.4/U in HIV^−^ subjects). Almost 100% of the γδ IELs were CD8^−^ and they often expressed the Vδ1/Jδ1-encoded epitope (median 65.2%). HIV^+^ patients on highly active antiretroviral therapy only tended to have a lower ratio of γδ IELs (median 12.8%) than those receiving no treatment (median 14.3%) or 1 nucleoside analogue (NA) (median 23.5%) or 2 NAs (median 13.0%). This minimal variation among therapy groups, contrasting the treatment response of systemic and local adaptive immunity, harmonizes with the novel idea derived from animal experiments that γδ T cells are largely innate cells in first-line microbial defence.

## Introduction

There is currently consensus that developing a human immunodeficiency (HIV) vaccine will be essential to stop the global acquired immunodeficiency syndrome (AIDS) epidemic [1], but human trials based on parenteral immunization have yielded disappointing results. Therefore, the general opinion is that more basic science studies of HIV cell entry and mucosal immunology are required to boost the development of an efficacious vaccine [2], [3]. Perhaps induction of a mucosal secretory immunoglobulin A (IgA) antibodies together with a cytotoxic response in mucosal and systemic CD8^+^ T cells is what novel efforts should aim at [4], [5]. In addition, it may be possible to reinforce innate immune mechanisms to enhance mucosal protection.

T cells expressing the γδ T-cell receptor (TCR) are believed to be critical in immune regulation, tumour surveillance and primary immune responses. Studies of TCR-mediated selection of T cells in mice support the view that some γδ subsets are unconventional and positively rather than negatively selected on cognate self antigen [6], [7]. However, recent findings have revealed effector functions apparently reflecting a mix of innate programming and acquired plasticity [8]. More than 20 years ago we reported a striking increase of duodenal γδ intraepithelial lymphocytes (IELs) in coeliac disease [9], and a γδ CD8^+^ IEL subset in such patients has recently been shown to have attributes of regulatory cells – at least partly by secreting TGF-β upon NKG2A– HLA-E interaction with intestinal epithelial cells [10].

We have also reported an increased proportion of duodenal γδ IELs in patients with hypogammaglobulinaemia associated with mild to moderate intestinal villous atrophy [11] and in selectively IgA-deficient subjects without infections [12]. We found in similar studies of HIV^+^ patients that the duodenal γδ IEL proportion was strikingly increased but, notably, decreased to normal levels in terminal AIDS cases less than 7 months before death [13].

Here, we retrospectively studied alterations in γδ IELs by two-colour immunofluorescence staining in duodenal tissue sections from patients with late-stage HIV type 1-infection. We related the distribution of these IELs to the number of B cells (CD19^+^), T cells (CD4^+^ and CD8^+^) and β_2_-microglobulin (β_2_-M) in peripheral blood as well as to clinical manifestations, treatment combinations, and various stages of disease progression. We had the opportunity to study immune alterations in severely immunodeficient patients during periods of simple treatment (1991–96), often with 1 nucleoside analogue (NA) or 2 NAs, compared with highly active antiretroviral therapy (HAART) of more recent years. By observing the response of IEL subsets – particularly the γδ T cells – compared with adaptive immunity markers, we hoped to obtain more mechanistic insight into their *in vivo* functional nature in humans as most of our current understanding of these unique immune cells come from studies in mice.

## Results

### Distribution of γδ intraepithelial lymphocytes

**Figure 1:**
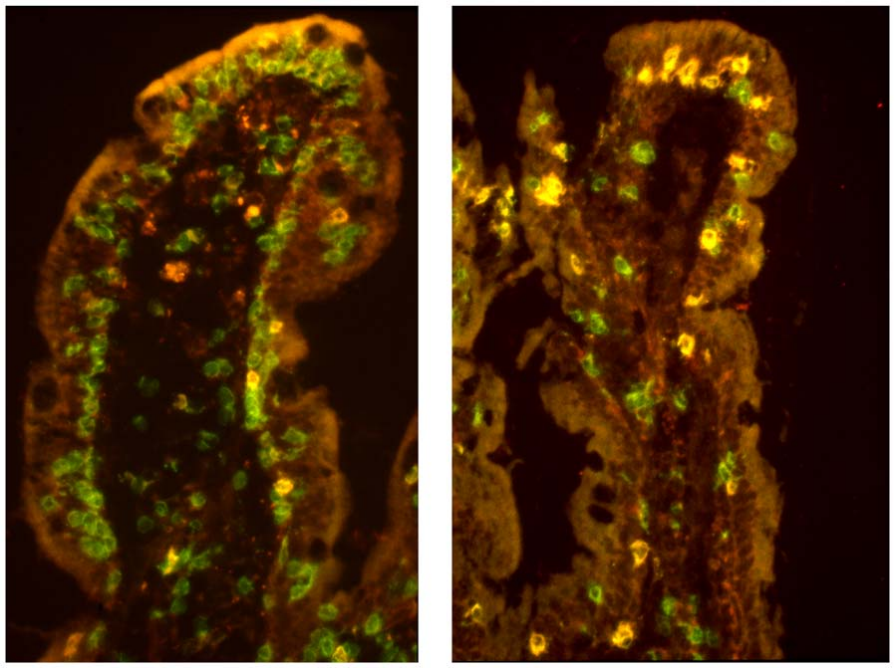
Paired immunofluorescence staining (merges) for CD3 (green) and γδTCR (red). The left panel shows a sectioned duodenal villus from an HIV^+^ patient who had retained an abundance of intraepithelial lymphocytes (IELs) which are mainly CD3^+^γδ TCR^−^ (presumably αβTCR^+^CD8^+^) and only few are CD3^+^γδTCR^+^ (yellow; median 14%). The right panel shows comparable field from an HIV^+^ patient with depletion of IELs but with highly increased CD3^+^γδTCR^+^ ratios (yellow; median 60%). In both sections some nonspecifically stained deposits are seen in the lamina propria, while the epithelium is visualized due to autofluorescence (which increased during storage of the frozen tissue samples).

**Figure 2:**
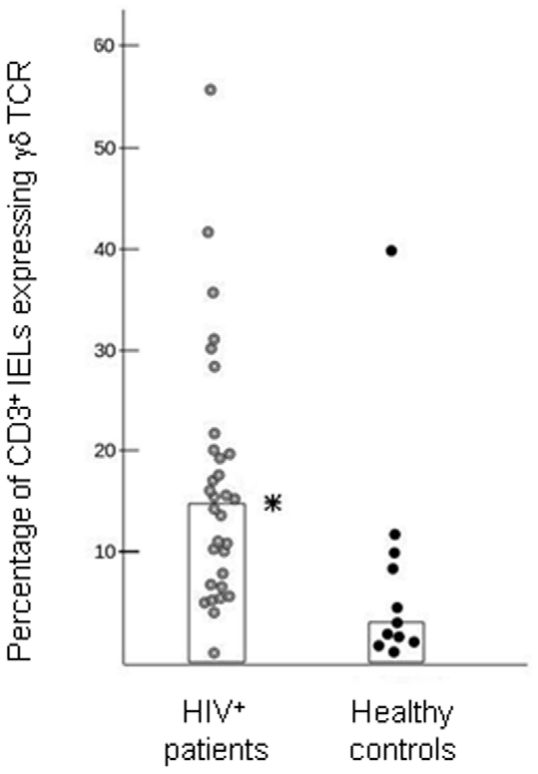
Scatter diagram of the percentage of duodenal γδ IELs in HIV^+^ patients compared with HIV^−^ control subjects. Median ratios are represented by vertical columns (*: p<0.02, Mann-Whitney two-tailed test).

The total number of CD3^+^ IELs per mucosal U was significantly lower (p<0.0001) in the HIV^+^ patients than in the controls (39.6/U *versus* 86.4/U). There was a striking variability among the patients as to the density of γδ IELs (Fig. 1) but the total number per mucosal length unit (U) tended to be increased (median 4.0/U *versus* 3.2/U). Therefore, the average γδ IEL ratio (Fig. 2) in the 30 HIV^+^ patients (median 14.5%, range 1.5–56.3%) was significantly increased (p<0.02) compared with values in the healthy controls (median 2.8%, range 0.3–38%). Autofluorescence in some of the old biopsies limited countable areas, and two patients were excluded from IEL counts/U. However, the ratios and numbers of γδ IELs were well correlated (*r* = 0.74), substantiating the validity of the recording methods.

**Figure 3:**
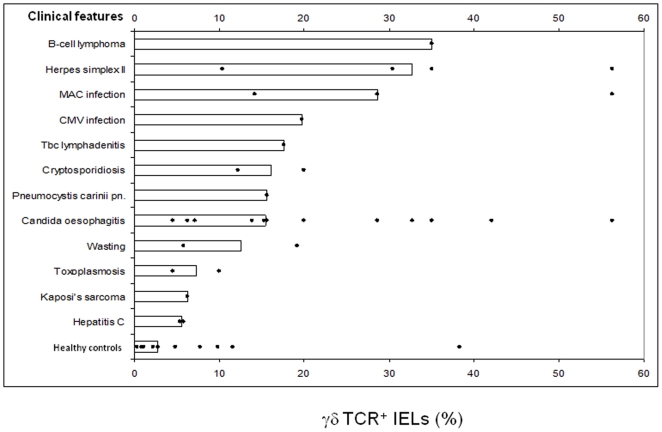
Scatter diagram of the percentage of duodenal γδ IELs in relation to clinical features of individual HIV^+^ patients. Median ratios are represented by horizontal columns. CMV, cytomegalovirus infection; MAC, *Mycobacterium avium intracellulare* complex.

Regarding the clinical subgroups of HIV^+^ patients, those with B-cell lymphoma, herpes simplex II infection, or with *Mycobacterium avium intracellulare* complex (MAC) infection had particularly high ratios of γδ IELs (Fig. 3). We also observed relatively high ratios in two patients with cytomegalovirus (CMV) infection or tuberculous lymphadenitis, whereas those with Candida infection showed a wide range (4.5%–56.3%). The lowest γδ IEL ratios were seen in HIV^+^ patients suffering from toxoplasmosis, Kaposi's sarcoma, or hepatitis C virus infection.

**Figure 4:**
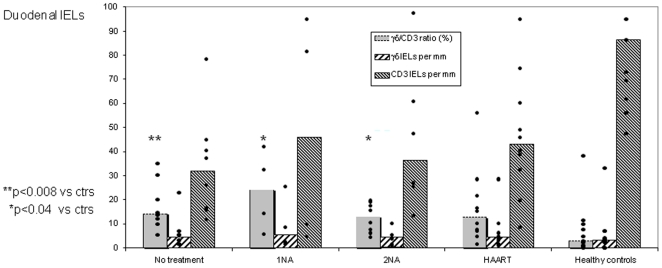
The γδ IEL subset remains elevated in AIDS patients despite antiretroviral treatment. Scatter diagram shows γδ∶CD3 ratios for duodenal IELs, γδ IEL numbers per mucosal unit (above 1 mm of muscularis mucosae), and numbers of CD3^+^ IELs per mucosal unit in HIV^+^ patients – all variables displayed according to administered antiretroviral treatment compared with no treatment and with data from HIV^−^ control subjects. Medians are represented by vertical columns. NA, nucleoside analogue; HAART, highly active antiretroviral therapy.

When we related the γδ IELs to different antiretroviral therapy, the highest ratios were seen in HIV^+^ patients on single medication (1 NA, median 23.5%; p<0.04 *versus* controls); these patients were the earliest included ones (before April, 1996) and often had intercurrent infections. The γδ IEL ratios were also increased in untreated patients (median 14.3%; p<0.008) and in those on double nucleoside analogues (2 NAs, median 13.0%; p<0.04), whereas those on triple combination therapy (HAART, median 12.8%) did not reach significance (p = 0.08) due to widely scattered ratios (Fig. 4).

As mentioned above, the total number of γδ IELs tended to be increased despite a highly significant decrease of the total number of CD3^+^ IEL/U in the HIV^+^ patients as a group (medians 31.7–45.7/U) compared with healthy controls (Fig. 4). In our previous studies we classificated three HIV^+^ subgroups according to length of survival after biopsy [13], [14], but this clinical grouping did not provide a better γδ IEL discrimination. It should be noted, however, that the 7 premortal patients died of diseases unrelated to AIDS, e.g. cerebral haemorrhagia, cardiac left ventricle dysfunction, aortic valve stenosis and neurolues.

Two-colour staining for CD8 revealed that almost all γδ IELs were CD8^−^ (median 100%, range 85–100%), as previously reported in coeliac disease (median 90%) [9] and primary immunodeficiencies (median 94–99%) [11], [12] and also in healthy controls (median 75%) [11]. A large fraction of γδ IELs in the HIV^+^ patients (median 65.2%, range 21.7–100%) expressed the Vδ1/Jδ1-encoded epitope revealed by mAb δTCS-1 – almost identical to that in patients with coeliac disease (67%) [9], primary immunodeficiencies (63–84%) [11], [12], and healthy controls (75%) [9], [11]. No difference was seen among the subgroups of HIV^+^ patients.

### Infection parameters in peripheral blood related to γδ IELs

When circulating T-cell subsets in HIV^+^ patients were compared with healthy blood donors [15], a tendency to reduced median numbers of CD8^+^ cells (0.36×10^9^/l; range, 0.057–1.67×10^9^/l *versus* 0.405×10^9^/l; range, 0.18–1.23×10^9^/l) was observed while the CD4 counts were, as expected, significantly (p<0.0001) reduced (0.020×10^9^/l; range, 0.0012–0.25×10^9^/l *versus* 0.815×10^9^/l; range, 0.5–1.58×10^9^/l) – particularly in late stage (Group II, 0.019×10^9^/l) and preterminal (Group III, 0.015×10^9^/l) AIDS patients.

Plasma HIV RNA (*n* = 12) increased as expected towards the end of life: Group I, 57000 copies/ml; Group II, 70000 copies/ml; and Group III, 191000 copies/ml. We observed a trend towards a higher CD4 count (0.060×10^9^/l, *n* = 9 *versus* 0.022×10^9^/l) and a lower median plasma HIV RNA level (76000 copies/ml) in patients receiving HAART.

No relationship was seen between the relative (Fig. 2) or absolute (Fig. 4) numbers of γδ IELs and the level of circulating CD4^+^ or CD8^+^ T cells, the CD4∶CD8 ratio or the B-cell number in peripheral blood.

### Serum immunoglobulins and β_2_ microglobulin

Hypergammaglobulinaemia was seen in 16 of the HIV^+^ patients, mostly IgG (18.3 g/l; range, 8.7–44.6 g/l) dominated by IgG1 (median, 11.2 g/l) and IgG3 (median, 1.8 g/l). There was only a slight increase of IgA (median 3.5 g/l) and also IgM (median, 1.5 g/l) was within normal limits. Compared with IgA in untreated HIV^+^ patients (median, 4.2 g/l), the level tended to be decreased in patients on HAART (median, 3.1 g/l), similarly to what we observed for duodenal IgA-producing plasma cells [14].

The β_2_-M level in HIV^+^ patients (median, 3.7 mg/l; *n* = 28) was significantly increased (p<0.0001) compared with healthy controls (median, 1.2 mg/l). No relationship was observed between serum β_2_-M and circulating CD4^+^ or CD8^+^ cells, duodenal γδ IELs or any of the other immunological variables.

### Long-time survivors

Three of the ten ARC/AIDS patients were so-called long-time survivors (LTSs), living for 15–17 years after becoming HIV^+^. Only one of them received HAART and one was not treated at all. Two of them were IVDAs and one was homosexual. Compared with the HIV^+^ patients as a group, they had lower median ratios and numbers of duodenal γδ IELs (7.1%, number 1.6/U), average levels of circulating CD4^+^ T cells, and mostly low serum IgG1. One 38-year old female IVDA received stavudine (d4T) and methadone. She suffered from wasting syndrome, AIDS dementia, and chronic hepatitis B and C virus infection, and had remarkably elevated serum immunoglobulins (especially IgG, 33.6 g/l) and relatively many duodenal IgG-producing plasma cells (15.2%), mainly of the IgG1 subclass [14]. We found elevated duodenal γδ IELs in this LTS (19.2%, number 5.5/U). All other immunological variables were near the average values.

## Discussion

More information is needed about the compartmentalized distribution of lymphoid and accessory immune cells in human intestinal mucosa to enable modulation of local immunity in a rational manner [16]. This has been emphasized in face of the failure of parenteral vaccines to reinforce immunity at the site of HIV entry [1]–[5]. However, ethical restrictions in sampling mucosal tissue from AIDS patients prohibit functional studies of local immune cells *ex vivo*, thus limiting our efforts to obtain mechanistic insight to observing their treatment response *in vivo*.

Here, we confirmed the relative increase of duodenal γδ IELs in HIV patients, as seen in our previous study of patients included before 1996 [13]. A concurrent study did not find elevated γδ IELs in HIV^+^ patients, whether they had diarrhoea or not [17]. This discrepancy might have a technical origin as the study was partly based on flow-cytometric analyses of dispersed cells. We performed quantitative *in situ* cell phenotyping and enumeration with a careful morphometric approach. In our HIV^+^ patients especially high γδ IEL ratios were associated with intercurrent B cell lymphoma, herpes simplex II infection, MAC infection, CMV infection, and tuberculous lymphadenitis.

Our results suggested that a relative expansion of the γδ IEL fraction occurs in advanced AIDS at the same time as the total IEL population is reduced. Most human IELs are memory αβ CD8^+^ T cells [18], [19]; based on recently published immunization experiments (both in humans and mice) [20], their long-term residence at body surfaces, including the gut epithelium, may depend on repetitive inductive activity in peripheral lymphoid organs. It has been reported that viral control and early HIV disease progression is associated with a synchronous differentiation of the HIV-specific and total CD8^+^ memory cell populations [21]. However, as the peripheral immune organs with their CD4^+^ helper T cells deteriorate in AIDS, chronic infectious stimuli are apparently unable to maintain the dominating αβ CD8^+^ IEL population due to lack of T-cell homing to the intestinal mucosa [22]. Thus, there must be other rules for γδ IELs which are maintained or expanded in response to opportunistic micro-organisms, apparently with no direct HIV effect as antiretroviral treatment did not reduce the response. This was in contrast to the expanded IgA^+^ duodenal plasma cell population that was significantly decreased by HAART in virtually the same patient material [14].

The function of γδ IELs is probably not limited to first-line defence. In mice the activation of γδ T cells sometimes follows that of αβ T cells, and the γδ T cells may mainly contribute to immune regulation and tissue repair [23]. The common overlap of γδ TCR-directed pathogen-encoded epitopes with molecules expressed by “stressed” host cells has fuelled the view that γδ T-cell responses are driven by lack of tissue homeostasis (inflammation, cell damage or transformation) rather than by microbial challenges [23]. In mice, it has been shown that γδ T cells express the toll-like receptors TLR1 and TLR2, as well as dectin-1, and respond to microbial products [24]. In addition, they express the aryl hydrocarbon receptor (AhR) and can therefore receive other environmental signals that may shape their functional capacity, including the production of the IL-17 cytokine [24]. Thus, these cells do not necessarily need TCR engagement but can be triggered by their innate receptors or, alternatively, by the cytokines IL-1 or IL-23 derived from activated macrophages or dendritic cells [25].

If it can be shown that human γδ IELs have immune modulating properties similar to murine γδ T cells, this would fit with the fact that they are not negatively affected by deterioration of the adaptive immune system in advanced AIDS, but rather expand in such HIV^+^ patients. Interestingly, it was recently shown that murine γδ IELs are a self-renewing population with little migration and recirculation out of their specialized niche, whereas peripheral γδ IELs are highly motile [26].

We have earlier reported a marked decrease of duodenal γδ IELs in premortal AIDS patients [13]. A combined deficiency of γδ IELs and IgA-producing plasma cells at the end stage of AIDS could in part be responsible for the fatal result. In the present study we could not show this; but it should be noted that most of the preterminal patients included here died of diseases unrelated to AIDS. There was a strikingly variable positive effect of HAART on the γδ IEL counts in the AIDS patients, although immune reconstitution was usually confirmed by favourable CD4-cell counts and plasma HIV RNA levels in peripheral blood.

Serum levels of β_2_-M and neopterin (an intermediate in the biosynthesis of tetrahydro-biopterin), which are markers of immunostimulation [27], showed an inverse correlation with duodenal γδ IELs in end-stage AIDS in our previous study [13]. A similar strong tendency of increasing β_2_-M concentrations towards the end of life was not observed in the present study. This could reflect the beneficial effect of HAART in advanced AIDS. One of the three LTSs showed particularly good prognosis (undetectable HIV RNA level) and had elevated serum IgG and duodenal IgG^+^ plasma cells (especially IgG1). This suggested complement-activating local immunity – a response also noted in other studies [28], [29]. Additionally, we found raised ratio and number of γδ IELs (19.2%, 5.5/U) in this patient, contributing to the idea that these cells exert immunoregulatory functions.

There is a recent focus on the intestinal epithelial barrier as an important variable in HIV infection. Pigtail macaques (PTMs) rapidly progress to AIDS after simian immunodeficiency virus (SIV) infection. The explanation seems to be that uninfected PTMs commonly show damage to their intestinal barrier [30]. This results in microbial translocation and deleterious immune activation both locally and systemically; a higher frequency of CD4^+^ memory/effector T cells ensues, and these cells are the primary target for SIV/HIV infection.

Th17 cells are important in microbial defence by attracting neutrophils and maintaining the mucosal barrier; but these CD4^+^ cells are preferentially lost during HIV infection [31], although they may be restored by HAART [32]. Importantly, however, γδ T cells may respond more rapidly with IL-17 secretion because of their innate nature [24], [25], [32], and therefore be compensating for the Th17 cell loss. Moreover, there is experimental evidence in mice that γδ T cells may amplify the function of Th17 cells [25] and inhibit regulatory cells T (T_reg_) cells [33]. Putative T_reg_ cells (CD4^+^CD25^+^FOXP3^+^) in peripheral blood may be increased in HIV^+^ patients and dampen various defences against the virus; but decrease of T_reg_ cells has been reported in chronic HIV infection [34], [35]. Thus, it remains unresolved whether T_reg_ cells are protective or detrimental in HIV^+^ patients. Also the balance between Th17 cells, γδ T cells and T_reg_ cells shows a complexity afflicted with many uncertainties [36]. Nevertheless, a recent study reported fewer T_reg_ cells in rectosigmoid mucosal biopsies of HIV long-term “controllers” than “non-controllers” [37].

Taken together, it remains unknown to what extent these immunological variables influence the course of HIV infection. However, γδ T cells with specificity for *Candida albicans*, and bearing the Vδ1 TCR chain as well as possessing cytoplasmic IL-17, have been identified in peripheral blood of HIV^+^ patients [38]. Whether these cells have similar functional properties as γδ IELs remains uncertain. We observed a relative expansion of duodenal Vδ1^+^ γδ IELs in AIDS patients, even in those receiving antiretroviral therapy. This probably reflected an intensified innate response to an increased luminal load of microbial components. Secondary intestinal infections are common in AIDS, and it is important to define how the mucosal defence behaves in these patients. A better delineation of intestinal HIV immunology, including the late stage-induced local response, is crucial to this end.

## Materials and Methods

### Biological samples

Duodenal biopsy specimens and peripheral blood were collected consecutively during the period 1991–98 from 30 patients infected with HIV type 1 (5 women: median age, 33 years and range, 26–40 years; and 25 men: median age, 39 years and range, 26–52 years); 18 were homosexual men, 7 intravenous drug abusers (IVDAs), and 5 had experienced heterosexual virus transmission in Africa (Table S1). Informed verbal consent was obtained from all patients. The biopsy material was derived from the Diagnostics and Treatment Biobank of the Oslo University Hospital. This collection of biological material intended for clinical examination, diagnostics and treatment does not require explicit informed consent according to The Norwegian Treatment Biobank Act 2003.

In addition, similar biopsy and blood samples were obtained from 11 immunologically intact HIV^−^ controls (seven females and four males; median age 21 years and range, 4.5–76 years) with no histological abnormalities in duodenal mucosa. Dyspepsia, growth retardation, irritable bowel syndrome, and hypothyroid disease were the reasons for endoscopic examination in a minority of the subjects as we also included volunteers (health workers) with no symptoms. Part of the same control material has been used in previous studies with informed written consent [11]–[13], and the Regional Ethics Committee approved the inclusion of additional controls to replace exhausted material.

All HIV^+^ patients had serum IgG antibodies to HIV type 1 as determined by ELISA (Organon Teknika, Boxtel, The Netherlands; or Abbott, Wiesbaden-Delkenheim, Germany) and this was confirmed by Western blotting (DuPont, Wilmington, DE). When clinically classified according to the criteria of the Centers for Disease Control and Prevention (CDC), Atlanta (GO, USA), 5 subjects were staged in group CDC IVA/B, 23 in group CDC IVC1, and 2 in group CDC IVD – the latter 25 fulfilling diagnostic criteria for full-blown AIDS. Because most of the patients were classified as having late-stage AIDS, CD4^+^ T-cell counts did not differ much. Median survival time after the first positive HIV test was 81 months (range 11–156 months; *n* = 20). Seven patients could retrospectively be defined as premortal due to death within 7 months (six homosexual men, one raped female refugee). However, they died mainly of diseases unrelated to AIDS, e.g. cerebral haemorrhagia, cardiac left ventricle dysfunction, aortic valve stenosis and neurolues.

### Clinical features

All HIV^+^ patients suffered from different on-going opportunistic and/or non-opportunistic infections; only one of them had skin or mucosal lesions consistent with Kaposi's sarcoma, and one had non-Hodgkin malignant lymphoma. Eight of the patients had chronic intermittent diarrhoea, of which two had cryptosporidiosis, one systemic CMV infection, and four herpes simplex type II infection. Altogether twelve patients had oral and/or oesophageal candidiasis, three suffered from MAC infection and two from wasting (>10% unwanted weight loss). Only one of these patients had duodenitis with histological signs of moderate, chronic inflammation. Other serious infections such as *Pneumocystis carinii* pneumonia was found in one patient, two had toxoplasmosis (encephalitis), two chronic hepatitis C virus infection and one tuberculous lymphadenitis (Table S1).

Four patients still received antiretroviral monotherapy with zidovudine (ZDV) at the time of sampling (1995–96) while eight used two other NAs such as didanosine (DDI) and/or stavudine (d4T) as well (Table S1). Ten patients tolerated satisfactorily combination therapy with one of these drugs together with lamivudine (3TC) and HIV protease inhibitors (PI) such as indinavir (Crixivan) or nelfinavir (Viracept), constituting HAART, whereas eight others had discontinued all antiretroviral treatment because of intolerable side effects. None received immunoglobulin replacement therapy.

### Quantification of Ig isotypes, B cells, T-cell subsets, β_2_-microglobulin and HIV RNA in peripheral blood

Measurements of serum IgG, IgA, and IgM were performed by nephelometry with commercial standards (Behringwerke, Marburg, Germany), while quantification of serum IgG subclasses was performed with monoclonal antibodies (mAbs) in commercial kits (The Binding Site, Birmingham, UK). The blood samples were collected as near the time of biopsy as possible (usually within 1 to 2 hours before) and analysed on the same day, or frozen at −20°C or −70°C for later analyses.

CD19^+^ B cells and CD4^+^/CD8^+^ T cells in peripheral blood were enumerated by a standard flow cytometric method. β2-M was measured by a microparticle enzyme immunoassay (Abbott IMX; Abbott laboratories, Abbott Park, IL, USA). HIV RNA was measured by a reverse transcriptase-polymerase chain reaction (RT-PCR) assay (Amplicor HIV-1 Monitor, Roche Diagnostic System, Brachburg, NJ, USA). The detection limit of the kit was 400 HIV-1 RNA copies/ml; lower values were also included. This methodology was only available after August 1996 in our hospital.

### Tissue sample processing

Multiple mucosal biopsy specimens (*n* = 8–10) were obtained endoscopically (Olympus GIF K10) from the distal duodenum. Endoscopical signs of chronic inflammation were occasionally observed in the distal oesophageal (*n* = 3), prepyloric gastric (*n* = 1) or duodenal mucosa (*n* = 2). Two specimens from each subject were processed for conventional histological evaluation. Other tissue specimens were fixed in 2% (w/v) paraformaldehyde for electron-microscopic evaluation or processed by snap-freezing in liquid nitrogen for immunohistochemistry (see below). All gastrointestinal endoscopies were performed in the HIV^+^ patients solely for clinical indications, mostly to evaluate their symptoms such as dysphagia, diarrhoea or cachexia. Ethical reasons prevented us from obtaining additional biopsies.

### Histological evaluation

A tissue section from each biopsy series was stained with haematoxylin and eosin for histological evaluation. In addition, scanning electron microscopy was performed to classify villus changes as previously described [11]. Such combined evaluation showed normal villi in all 30 HIV^+^ patients. Only two of them had histological signs of moderate, chronic duodenal inflammation. All control subjects had histologically normal duodenal biopsies.

### Two- or three-colour immunohistochemical staining

Serial cryosections were cut at 6 µm and incubated for 1 hour at ambient temperature with pairs of unlabelled murine mAbs of the following human specificities: δ chain of γδ TCR (TCRδ1, IgG1, 1/20; T Cell Sciences Inc., Cambridge, MA, USA) and CD3 (clone RIV9, IgG3, 1/10; Sanbio, Am Uden, The Netherlands); δ chain of γδ TCR and the α chain of CD8 (BMA-081, IgG2a, 1/40; Behringwerke, Marburg, Germany); CD4 (anti-Leu-3a and -3b, IgG1, 1/40; Becton Dickinson, Mountain View, CA, USA) and CD3 (RIV9, IgG3, 1/10; Sanbio); and CD8 (anti-Leu-2b, IgG2a, 1/20; Becton Dickinson) mixed with BMA-081 (IgG2a, 1/80) and CD3 (anti-Leu-4, IgG1, 1/80; Becton Dickinson). Secondary antibody reagents were various combinations of biotinylated and fluorescein isothiocyanate-conjugated goat IgG (0.01 to 0.05 g/litre) with specificity for mouse IgG1, IgG2a, or IgG3 (Southern Biotechnology, Birmingham, ALA, USA). These reagents were applied for 1.5 hour, and then streptavidin-Texas red (0.0025 g/litre; Bethesda Research Laboratories, Gaithersburg, MD, USA) was applied for 30 min. Human IgG at 0.8 g/litre (Kabi Vitrum, Stockholm, Sweden) was added to the goat reagents to block species cross-reactivity [9]. Because mAb TCRδ1 reacts with all γδ T cells, sequential staining was performed as detailed elsewhere [9] to examine the subset expressing the Vδ1/Jδ1-encoded epitope for mAb δTCS-1 (IgG1; T Cell Sciences).

### Fluorescence microscopy and cell counting

Immunofluorescence was examined at a magnification of ×300 in a Nikon microscope (Eclipse E600; ocular grid; Nikon, CFI) equipped with a Ploem-type vertical illuminator with interference filters for selective observation of red, green, or blue emission. The results were recorded on Ectachrome professional 800/1600 ASA daylight film. All sections were examined blinded by the same investigator. The percentage distribution of γδ IELs was determined by evaluating every CD3^+^ IEL for γδ TCR expression (Fig. 1). Also coexpression of CD8 and Vδ1/Jδ1 on γδ IELs was similarly examined.

The total number of CD3^+^ and γδ IEL was related to a 316-µm grid width, including all parts of the epithelium from the luminal face to the muscularis mucosae. The number of CD3^+^ and γδ IELs in four such defined parallel areas was determined and calculated to be expressed per millimetre of muscularis mucosae (mucosal length unit, U). When necessary, cytokeratin immunostaining was used to outline the epithelial compartment; a rabbit antiserum (1/100) [39] was followed by 7-amino-4-methylcoumarin-3-acetic acid-conjugated goat anti-rabbit IgG (1/20; Vector Laboratories, Burlingame, CA, USA).

### Control reference data

The ratios of γδ IELs as well as the numbers of CD3^+^ and γδ IELs per mucosal U were determined as described above in histologically normal duodenal mucosa of the eleven HIV^−^ control subjects. Reference data were available in our laboratory for the normal expression of the Vδ1/Jδ1-encoded epitope on γδ IELs [9]. Normal CD4∶CD8 T-cell ratios for duodenal lamina propria and epithelial compartments were based on published data [40].

As normal reference data for circulating lymphocyte subsets, we used results from healthy (HIV^−^) blood donors (*n* = 60) [15] recorded as described previously [41]. Reference data from blood donors were also available for serum concentrations of β_2_-M (*n* = 18). The HIV RNA level in normal controls was set at <400 copies/ml plasma.

### Statistical analysis

Statistical calculations were based on the software program SPSS (Chicago, IL) while the Mann-Whitney 2-tailed test for unpaired samples was used for comparison between various categories of HIV^+^ patients and control subjects. Results are presented as median and observed range. Differences between groups were considered statistically significant at p<0.05. Correlations between mucosal CD3^+^ IELs or γδ IELs and circulating T-cell subsets or β_2_-M in the HIV^+^ patients and the HIV^−^ controls were calculated by the Spearman rank correlation test (SPSS), as was the relationship between the proportions and numbers of γδ IELs. The reproducibility of *in situ* T-cell enumerations by paired immunofluorescence staining was excellent as documented in our previous studies [9], [11], [12].

### Ethical considerations

The study was approved by the Committee for Medical Research Ethics (Health Region South-East, Oslo, Norway).

## Supporting Information

Table S1 **Clinical and immunological features of the HIV-1-infected patients in relation to treatment regimen.**
(DOC)
