# Supplementary material for: Intraepithelial γδ T Cells Remain Increased in the Duodenum of AIDS Patients Despite Antiretroviral Treatment
Source: PLoS One. 2012 Jan 4;7(1):e29066. doi: 10.1371/journal.pone.0029066 (PMC3251554; doi:10.1371/journal.pone.0029066)
Supplement: Table S1 — Clinical and immunological features of the HIV-1-infected patients in relation to treatment regimen. (DOC) [file pone.0029066.s001.doc]

**Table S1.** Clinical and immunological features of the HIV-1-infected patients in relation to treatment regimen

| **Patients, sex, age (yrs.)** | **Anti-HIV regimen**  **and duration** | **Current infections** | **Current**  **antibiotics** | **Other serious diseases** | **Blood CD4^+^ T cells (10^9^/l)** | **Plasma HIV-RNA (copies/ml)** | **γδ IELs: %/**  **nos. per unit** |
| --- | --- | --- | --- | --- | --- | --- | --- |
| 1. M, 44 | None | Herpes oesophagitis,  oral candidiasis | Fluconazole | Non-Hodgkin’s lymphoma later | 0.01 | ND* | 30.4/23 |
| 2. M, 38 | None | Cryptosporidiosis | None |  | 0.02 | ND | 12.2/3.2 |
| 3. M, 31 | None | Candida oesophagitis, cryptosporidiosis | Fluconazole |  | 0.03 | ND | 20/3.2 |
| 4. M, 39 | None | Candida oesophagitis | Fluconazole, TMP/SMX | Ethanol abuser | 0.008 | ND | 13.9/5.5 |
| 5. M, 26 | None | Oral candidiasis,  toxoplasmosis | Fluconazole, TMP/SMX | Hepatitis C, cerebral lesions | 0.009 | ND | 10/1.6 |
| 6. M, 38 | None | Candida oesophagitis, pneumonia, genital herpes | Clindamycin, Tobramycin | Non-Hodgkin’s lymphoma | 0.012 | ND | 35/5.6 |
| 7. M, 44 | None |  |  | Heroin abuser, hep. C | 0.132 | 130,000 | 5.4/1.6 |
| 8. M, 49 | None | Oral candidiasis | TMP/SMX, Nystatin | Wasting | 0.05 | 57,000 | 14.7/7.1 |
| 9. M, 34 | 1 NA, 35 mths. | Systemic Candida infection, UTI, MAC and PCP infection | TMP/SMX, Ethambutol, Clarithromycin, Ketokonazole | Cerebral haematoma | 0.001 | ND | 42.1/8.7 |
| 10. F, 40 | 1 NA, 6 mths. | Candida oesophagitis | Fluconazole, TMP/SMX, Ethambutol, Azithromycin | Heroin abuser | 0.01 | ND | 32.7/1.6 |
| 11. M, 34 | 1 NA, 10 mths. |  |  | Heroin abuser,  hepatitis C, wasting | 0.22 | ND | 5.8/2.4 |
| 12. F, 26 | 1 NA, 8 mths. | MAC | Nystatin, Acyclovir  Ketokonazole |  | 0.02 | ND | 14.2/25.4 |
| 13. M, 38 | 2 NA, 5-7 days | Candida oesophagitis,  MAC | TMP/SMX, Fluconazole, Doxycyclin | Heroin abuser | 0.012 | 70,000 | 7.7/4.0 |
| 14. F, 38 | 2 NA, 7 weeks |  | TMP/SMX | Heroin abuser, wasting, hepatitis B+C, AIDS dementia | 0.114 | ND | 19.2/5.5 |
| 15. M, 48 | 2 NA, 8 mths. | Pleuritis, herpes oesophagitis | TMP/SMX | Stenosis valvula aortae | 0.132 | 1,400 | 10.4/10.3 |
| 16. M, 28 | 2 NA, 14 days | PCP infection,  oral candidiasis | TMP/SMX, Fluconazole | Oesophageal ulcers | 0.002 | ND | 15.6/0.8 |
| 17. M, 29 | 2 NA, 18 mths. | Oral candidiasis, toxoplasmosis | TMP/SMX | Wasting, cerebral lesions | 0.015 | ND | 4.5/1.6 |
| 18. M, 42 | 2 NA, 3 mths. | Tuberculous lymphadenitis | TMP/SMX, Fluconazole, Ethambutol |  | 0.02 | ND | 17.6/0 |
| 19. M, 41 | 2 NA, 42 mths. | Systemic CMV infection | Amphotericin B, Ketokonazole | Gastritis | 0.072 | ND | 19.7/0 |
| 20. M, 37 | 2 NA, 8 mths. | Candida oesophagitis | TMP/SMX | Kaposi sarcoma | 0.018 | ND | 6.3/4.0 |
| 21. M, 39 | HAART, 5 mths. | Pneumonia, Candida oesophagitis | TMP/SMX, Fluconazole | Heroin abuser,  hepatitis B+C | 0.01 | 16,000 | 5.0/1.6 |
| 22. M, 40 | HAART, 13 mths. | MAC | Clarithromycin, Ethambutol, Ciprofloxacin | Pulmonary bacillar angiomatosis | 0.19 | <400 | 21.6/6.3 |
| 23. F, 33 | HAART, 15 mths. | MAC, sinusitis, pelvic abscess | Ciprofloxacin, Ethambutol, Rifabutin |  | 0.14 | 380,000 | 16.7/28.5 |
| 24. M, 45 | HAART, 14 mths. | Systemic CMV infection | Clarithromycin, Ethambutol | Granulomatous hepatitis (side effect?) | 0.25 | 42,000 | 7.9/4.0 |
| 25. F, 32 | HAART, 6 days | Oral candidiasis | Fluconazole | Heroin abuser, wasting | 0.054 | 110,000 | 1.5/0.8 |
| 26. M, 44 | HAART, 10 mths. | Candida oesophagitis, gastritis | TMP/SMX | Wasting | 0.05 | >750,000 | 10.3/4.8 |
| 27. M, 43 | HAART, 3 mths. | Candida oesophagitis, MAC, genital herpes | Ethambutol, Rifampin, Clarithromycin, Ciprofloxacin, Acyclovir |  | 0.006 | ND | 56.3/28.5 |
| 28. M, 29 | HAART, 2 mths. | Candida oesophagitis | TMP/SMX, Fluconazole | Wasting | 0.009 | ND | 15.3/10.3 |
| 29. M, 52 | HAART, 3 mths. | Oral candidiasis, Candida oesophagitis, MAC | TMP/SMX, Fluconazole |  | 0.024 | <400 | 28.6/3.2 |
| 30. M, 39 | HAART, 8 days | Candida oesophagitis | Ketokonazole |  | 0.112 | 190,000 | 7.1/1.6 |

*Abbreviations: ND = not determined; TMP = Trimethoprim; SMX = Sulphamethoxazole; NA = nucleoside analogue; HAART = highly active antiretroviral therapy;

MAC = *Mycobacterium avium intracellulare* complex.
